# Peripheral Blood Cell Ratios as Prognostic Indicators in a Neoadjuvant Chemotherapy-Treated Breast Cancer Cohort

**DOI:** 10.3390/curroncol29100591

**Published:** 2022-10-07

**Authors:** Amirhossein Jalali, David Miresse, Matthew R. Fahey, Niamh Ni Mhaonaigh, Andrew McGuire, Emer Bourke, Michael J. Kerin, James A. L. Brown

**Affiliations:** 1 School of Medicine, University of Limerick, V94 T9PX Limerick, Ireland; 2 Health Research Institute (HRI), University of Limerick, V94 T9PX Limerick, Ireland; 3 Discipline of Surgery, Lambe Institute for Translational Research, School of Medicine, University of Galway, H91 TK33 Galway, Ireland; 4 Discipline of Pathology, Lambe Institute for Translational Research, School of Medicine, University of Galway, H91 TK33 Galway, Ireland; 5 Centre for Chromosome Biology, University of Galway, H91 TK33 Galway, Ireland; 6 Limerick Digital Cancer Research Centre (LDCRC), Bernal Institute, University of Limerick, V94 T9PX Limerick, Ireland

**Keywords:** breast, monocytes, lymphocytes, neutrophils, basophils, eosinophils, ratio, biomarker, NLR, LMR, NWR, LWR, MWR, immunocyte, peripheral, blood, cancer, neoadjuvant, NAC, systemic

## Abstract

Breast cancer represents a heterogeneous condition in which the interaction between host immune response and primary oncogenic events can impact disease progression. Ratios of systemic blood-based immunocytes have emerged as clinically-relevant prognostic biomarkers in cancer patients. The NLR (neutrophil-to-lymphocyte ratio) has been shown to be prognostic in a variety of cancers, including breast cancer. However, evaluation of the prognostic value for overall survival (OS) and disease-free survival (DFS) of other key immunocyte ratios—neutrophil-to-lymphocyte ratio (NLR), lymphocyte-to-monocyte ratio (LMR), neutrophil-to-white cell count ratio (NWR), lymphocyte-to-white cell count ratio (LWR), monocyte-to-white cell count ratio (MWR), platelet-to-lymphocyte (PLR)—by breast cancer subtypes in a neoadjuvant chemotherapy (NAC) cohort remains to be fully explored. An NAC-treated breast cancer cohort, comprised of Luminal A, Luminal B, HER2-positive, and triple negative/basal breast cancers, treated at a tertiary referral center (minimum 3-year follow-up), was used to calculate immunocyte ratios and immunocyte cut-off values, calculated with >80% specificity (using decision tree modeling). The association with subtype-specific OS, DFS, and tumor grade was analyzed using cut offs calculated using both receiver operating characteristic curves and decision tree modelling. Decision tree calculated ratios showed that LMR (5.29) and MWR (0.06) were significantly associated with Luminal A OS (*p* = 0.004 and *p* = 0.022) and DFS (*p* = 0.004 and *p* = 0.022), and Luminal B OS (*p* = 0.027 and *p* = 0.008) and DFS (*p* = 0.005 and *p* = 0.007). NLR (1.79) and LWR (0.30) were significantly associated with HER2-positive OS (*p* = 0.013 and *p* = 0.043). NLR (1.79) and NWR (0.62) were significantly associated with DFS (*p* = 0.035 and *p* = 0.021). No significant association we observed between any immunocyte ratio in the triple negative cohort. Our results demonstrate the subtype-specific prognostic value of immunocyte ratios in NAC-treated breast cancer patients. Further validation of immunocyte ratios will provide clinicians with a new prognostic aid for disease management and monitoring.

## 1. Introduction

Inflammation, including low-level chronic inflammation, is associated with several diseases, including many cancers [1,2]. Systemic inflammation can be characterised through the quantification of peripheral blood cell types and counts (including neutrophils, lymphocytes, platelets and monocytes). Quantitative levels (numbers) of these peripheral immune cells (immunocytes) can be made from routine blood samples collected as part of the standard disease diagnostics and management. Inflammation associated with tumourigenesis in solid tumours can be mediated by the presence of Leukocytes (including monocytes, lymphocytes, neutrophils, basophils and eosinophils), facilitating their use as biomarkers [2,3,4]. 

Breast cancer represents a heterogeneous condition in which the interaction between host immune response and primary oncogenic events can impact disease progression. Systemic inflammatory marker ratios, predominately the neutrophil lymphocyte ratio (NLR), have been investigated as prognostic markers for many diseases [5,6,7]. The neutrophil-to-lymphocyte ratio (NLR) has emerged as a clinically-relevant measure of immune function in tumours, including breast cancer [8,9,10,11]. Ratios of peripheral blood cells have been correlated with clinically relevant outcomes, such as disease-free survival (DFS) and overall survival (OS) in breast cancer [12,13].

Previous work using a large and unstratified American breast cancer cohort demonstrated that an NLR >3.3 was predictive of the 5 year patient survival rate [7]. It has been shown that a low pretreatment NLR in neo-adjuvant chemotherapy (NAC) treated breast cancer patients is associated with pathological complete response (pCR) [9,10]. Furthermore, NLR has been identified as an independent predictor of relapse-free survival (RFS) in NAC treated patients [14]. Furthermore, investigating immunocyte ratios as prognostic marker in breast cancer molecular subtypes found that lymphocyte-monocyte ratio (LMR) were prognostic factors of DFS in Luminal A, Luminal B and Her positive tumours. In Triple negative breast cancer (TNBC) tumours the NLR predicted DFS [15].

While there are several studies investigating NLR in breast cancer, few studies have examined any other systemic inflammatory immunocyte marker ratios (Neutrophil to Lymphocyte ratio (NLR), Lymphocyte to Monocyte ratio (LMR), Neutrophil to White cell count ratio (NWR), Lymphocyte to White cell count ratio (LWR), Monocyte to White cell count ratio (MWR), Platelet to Lymphocyte (PLR)), as prognostic markers. Here in a single neoadjuvant chemotherapy treated breast cancer cohort we evaluate the prognostic value (Overall survival (OS) and Disease-free survival (DFS)) of all major immunocyte cell ratios (NLR, LMR, NWR, LWR, MWR and PLR) by major breast cancer subtypes (Luminal A, Luminal B, Her2 positive and Triple negative/Basal). We further advance our understanding of the use of immunocyte marker ratios by evaluating their relationship to tumour grade and to each other. 

## 2. Materials and Methods

### 2.1. Case Selection

A neoadjuvant chemotherapy-treated cohort was selected from a retrospective database (2006–2017) of histologically confirmed breast cancer (subtypes indicated) patients, collected consecutively from the tertiary referral center (Galway University Hospital). Blood counts were taken from blood collected prior to treatment. The details of individual cohorts of the neo-adjuvant chemotherapy treated patients are as follows: Luminal A (*n* = 108), Luminal B (*n* = 122), HER2-positive (*n* = 41), Triple negative/basal (*n* = 43) patients with a follow-up of at least 3 years.

### 2.2. Pathology

Analysis was performed as part of the routine clinical evaluation by clinical pathologists at the Pathology Laboratory, University Hospital Galway. ER and PR receptor status was determined using immunohistochemistry as per ASCO guidelines (ALLRED score > 2, or more than 1% stain positive). The HER2 receptor status was identified by HercepTest, with a score of 3+ considered positive. Any + 2 inconclusive results were confirmed using FISH testing as per ASCO guidelines, with a HER2/CEP17 ratio > 2 considered as amplified.

### 2.3. Ratio Calculations

The indicated systemic inflammatory marker ratios for each patient were calculated. The calculated systemic inflammatory marker ratio scores for each patient were correlated with indicated clinicopathological details (including age at diagnosis, overall survival, and disease-free survival).

### 2.4. Ethics Approval and Consent to Participate

The ethical approval for this study was granted by the University College Hospital Galway and the National University of Ireland Galway (C.A.151). All patient clinicopathological data was obtained from a prospectively maintained anonymized database.

### 2.5. Statistical Analysis

Statistical analysis was performed using R software (version 4.2.0). Descriptive statistics were performed by cancer subtypes. The Kruskal–Wallis test was used to examine the statistical significance of differences in the median of continuous variables between patients with Luminal A, Luminal B, HER2-positive, and triple negative breast cancer. An appropriate analysis from Pearson’s chi-squared test or Fisher’s exact test was used to investigate the significant differences between categorical variables. A *p*-value of <0.05 was considered statistically significant. Pearson correlations were estimated to investigate the relationship between each blood marker (immunocyte) ratio and 3-year overall survival (OS) and 3-year disease-free survival (DFS). The relationship between blood marker ratios and tumor grade were assessed using one-way analysis of variance. Receiver operating characteristic (ROC) cut-off points for each blood marker ratio were chosen so as to ensure at least 80% sensitivity for 3-year OS and 3-year DFS, and the corresponding specificity was calculated. The optimal ROC cut-off points were chosen by applying decision tree methods, and log-rank tests were carried out to investigate the statistical difference between low- and high-blood marker ratios.

### 2.6. Multivariate Analysis and Modeling

Cox regression model [16] was used to investigate the multivariate effect of blood marker ratios and the clinical variables on OS and DFS. Excluding 21 patients with tumor grade 0 and 1, the logistic regression model is used to investigate the role of blood marker ratios and the clinical variables in distinguishing between tumor grade 2 or grade 3. The stepwise selection technique was applied to identify the best blood marker ratio(s). The model discrimination was demonstrated using the ROC curve (at 3 years, for OS and DFS models) by plotting the sensitivity and specificity of the model at each of its risk thresholds. Internal validation was built into the cross-validation approach to prevent overfitting of the data by using a 10-fold cross-validation. Calibration plots were generated to assess the agreement between the observed incidence of cancer and predicted risk [17].

## 3. Results

### 3.1. Patient Demographics

A neo-adjuvant chemotherapy-treated cohort of 312 breast cancer patients was formed, consisting of Luminal A (*n* = 108), Luminal B (*n* = 122), HER2-positive (*n* = 41), and Triple negative/basal (*n* = 43) patients with a follow-up of at least 3 years (Table 1).

In the Luminal A subtype, 23.1% (*n* = 25) suffered a recurrence, and 20.4% (*n* = 22) died of breast cancer. In the HER2-positive subtypes, 11% (*n* = 18) suffered a recurrence, and 13.5% (*n* = 22) died of breast cancer. In the TNBC subtype, 11.6% (*n* = 5) suffered a recurrence, and 16.3% (*n* = 7) died of breast cancer.

In the total cohorts, the median age at diagnosis was 55 years old, with a median overall survival (OS) of 45 months, and median disease-free survival (DFS) of 40 months. The predominant histologic grade for the cohort was grade 3 (53.2%, *n* = 167), followed by grade 2 (41.4%, *n* = 130) (Table 1). A total of 40.5% (*n* = 137) of patients received neo-adjuvant chemotherapy.

Examining the immunocyte count in the patient samples collected prior to treatment (as a collective cohort, and by individual breast cancer subtype), we found that the only significant difference was observed in the neutrophil (*p* = 0.025) and eosinophil (*p* = 0.023) counts (Table 2).

### 3.2. Systemic Inflammatory Marker Ratios

**Figure 1:**
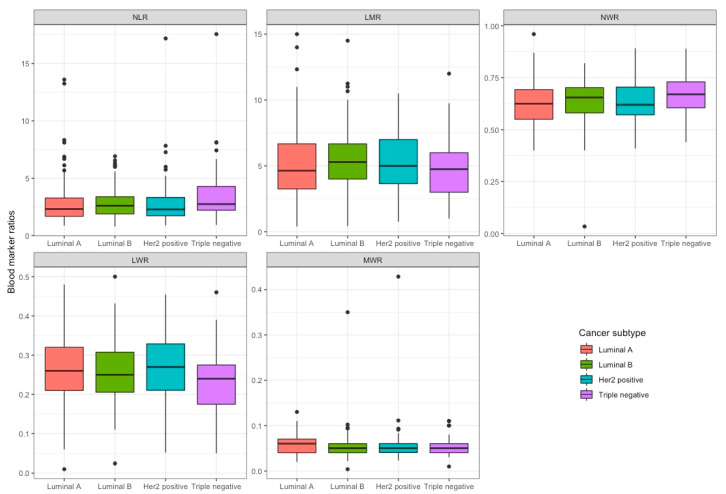
Box plots of immunocyte ratios by indicated breast cancer subtype.

We then calculated the median immunocyte ratios in the patient samples, by complete cohort and by individual breast cancer subtype. We calculated the neutrophil to lymphocyte ratio (NLR), lymphocyte to monocyte ratio (LMR), neutrophil to white cell count ratio (NWR), lymphocyte to white cell count ratio (LWR), monocyte to white cell count ratio (MWR), and platelet to lymphocyte (PLR). We found that there was no significant difference between any of median immunocyte ratios of any of the groups examined (Figure 1 and Table 3).

### 3.3. Correlating Clinicopathological Details with Systemic Inflammatory Marker Ratios

To explore the prognostic utility of the median immunocyte ratios, we examined the correlation between patients 3-year overall survival (OS), disease-free survival (DFS), and tumor grade. To further stratify the cohort, we examined these factors by breast cancer subtype (as well as by combined cohort) (Table 4, Supplementary Table S1).

In the Luminal A subtype, a higher MWR is significantly associated with poorer survival outcome for both OS (*p* = 0.01) and DFS (*p* = 0.018). In Luminal B patients, a lower LMR is significantly associated with poorer survival outcome for both OS (*p* = 0.018) and DFS (*p* = 0.005), and a higher MWR is significantly associated with poorer survival outcome for both OS (*p* = 0.014) and DFS (*p* = 0.009). In the HER2-positive subtype, a higher NLR is significantly associated with poorer survival outcome for OS (*p* = 0.018) and DFS (*p* = 0.006), a higher NWR is significantly associated with poorer survival outcome for OS (*p* = 0.005) and DFS (*p* = 0.001), and a lower LWR is significantly associated with poorer survival outcome for OS (*p* = 0.006) and DFS (*p* = 0.003). There was no significant correlation between immunocyte ratio and both OS and DFS in the triple negative subtype. No significant correlation was observed between any immunocyte ratio and subtype stratified tumor grade.

Examining breast cancer as a combined cohort, we found that a higher NLR is significantly associated with a poorer DFS (*p* = 0.038), and a higher MWR is significantly associated with a poorer OS (*p* = 0.005) and DFS (*p* = 0.015) survival outcome. Interestingly, only when evaluating the combined breast cancer cohort did we find a significant association between tumor grade and an immunocyte ratio, or the NWR (*p* = 0.029) (Table 4).

### 3.4. Selected Cut-Off Values for Immunocyte Cell Ratios

A receiver operating characteristic (ROC) curve of the OS was created to determine the optimal cut-off points (set to ensure >80% sensitivity) of the immunocyte ratios. The threshold cut-off values of each of the immunocyte ratios, calculated for each breast cancer subtype and the combined cohort, are shown in Table 5.

A ROC curve of the 3-year DFS was created to determine the optimal cut-off points (set to ensure >80% sensitivity) of the immunocyte ratios. The threshold cut-off values of each of the immunocyte ratio, calculated for each breast cancer subtype and the combined cohort, are shown in Table 6.

Using decision tree analysis, the immunocyte ratio cut-off values calculated in Table 5 (OS) and Table 6 (DFS) are adjusted to optimize the difference between the two groups (low and high immunocyte values) with respect to OS (Table 7) and DFS (Table 8).

Using the calculated cut offs, in the Luminal A subtype, both the LMR (*p* = 0.004) and MWR (*p* = 0.022) were significantly associated with OS. The same significant association was observed for Luminal A DFS and LMR (*p* = 0.004), and MWR (*p* = 0.022).

Examining the association between Luminal B immunocyte cut offs and OS, LMR (*p* = 0.027) and MWR (*p* = 0.008) were significant. Again, the same significant association was observed for Luminal B DFS and LMR (*p* = 0.005), and MWR (*p* = 0.007).

A significant association was found between OS in the HER2-positive subtype and the immunocyte cut offs NLR (*p* = 0.013) and LWR (*p* = 0.043). When evaluating HER2-positive DFS using immunocyte cut offs, we found a significant association with NLR (*p* = 0.035) and NWR (*p* = 0.021).

There was no significant correlation between OS or DFS and immunocyte ratio cut off in the triple negative subtype. Additional multivariate modeling and analysis are presented in Supplementary Figure S1A,B.

By assessing both the OS and DFS of all breast cancer cases as a combined cohort using the immunocyte cut-off scores, we discovered a highly significant association between OS and LMR (*p* = <0.001) and MWR (*p* = 0.001). The inspection of DFS and immunocyte cut offs revealed a similar pattern of highly significant association between LMR (*p* = <0.001) and MWR (*p* = 0.004).

Based on the Cox models, LMR is identified to be the only marker ratio for OS (HR of 0.94). Further analysis indicates that integrating cancer subtypes into LMR would improve the model performance for predicting the risk of 3-year OS (Supplementary Figure S1A). LMR is also identified to be an important marker ratio for DFS (HR of 0.93), where integrating distal metastasis into LMR improved the model performance for predicting the risk of 3-year DFS (Supplementary Figure S1B).

**Figure 2:**
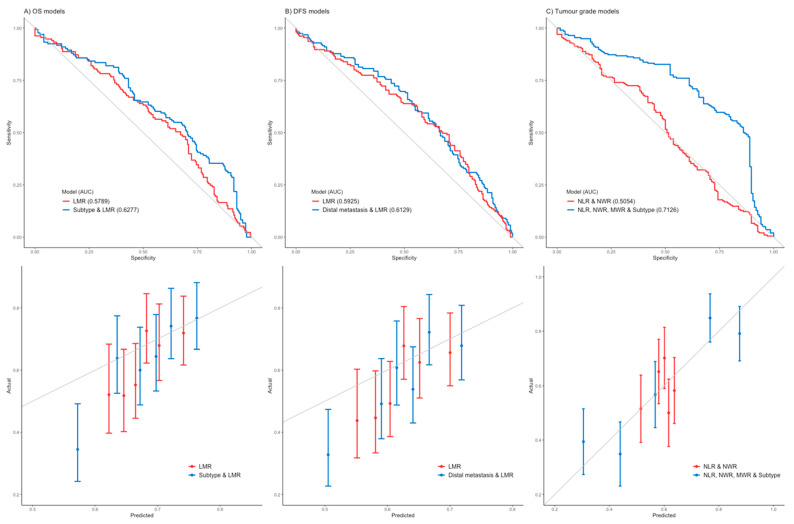
Multivariate model performance, top, and calibration, bottom. (**A**) Models for OS at 3 years, including LMR, and breast cancer subtype and LMR models. (**B**) Models for DFS at 3 years, including LMR, and distal metastasis and LMR. (**C**) Models for tumors grade at 3 years, including LMR and NWR, and NLR and NWR, MWR and breast cancer subtype models.

The result of the logistic regression shows that NLR (OR of 0.86) and NWR (OR of 25.6) are two important marker ratios for discriminating between tumors grades 2 and 3. However, further analysis indicates that integrating cancer subtype and MWR with NLR and MWR improved the discriminatory ability of the model (Figure 2).

## 4. Discussion

The use of immunocyte ratios holds the promise of harnessing routinely collected clinical data and applying it to calculate a clinically informative prognostic factor. Our results here have comprehensively catalogued and evaluated a range of immunocyte ratios (neutrophil to lymphocyte ratio, lymphocyte to monocyte ratio, neutrophil to white cell count ratio, lymphocyte to white cell count ratio, monocyte to white cell count ratio, and platelet to lymphocyte ratio) in a neoadjuvant treated breast cancer cohort. We advance the work defining the relationship between immunocyte ratios and NAC by calculating optimized cut-off values for each subtype and clinicopathological trait examined using a decision tree-based approach. We note that a potential limitation of our approach is in the analysis using a multiplicity of testing, which has the potential to inflate type 1 errors. However, many of the significant correlations found (Table 4) were also significant in the multivariate analysis (see Supplementary Figures and Tables). We suggest that the decision tree-based approach is a more relevant and tailored analysis approach, highlighting the differences between subtypes, immunocyte ratios, and responses.

NLR is one of the most commonly evaluated immunocyte ratio in breast cancer, and has often been shown to significantly associate with OS, DFS or pCR [6,7,8,9,11,12,13,14,15,18,19,20,21,22,23,24]. However, few studies have evaluated applying more than one single immunocyte ratio in their cohorts. Our subtype specific NLR ratio cut offs are within similar ranges from these studies, with these reported NLR cut off ratios ranging from between 1.26 to 2.74 [6,10,12,15]. The range of cut offs reported is likely indicative of intrinsic differences in the cohorts (such as age, menopause status, geographical location, ethnicity), in line with other differences associated with changes in breast cancer outcomes [25,26].

While our results further reinforce the prognostic value of the NLR, they also demonstrate that the range of the other immunocyte ratios evaluated (LMR, NWR, LWR, MWR) are equally relevant prognostic factors, demonstrating breast cancer subtype specificity in this NAC treated cohort.

Additionally, by providing the median values of each of the immunocyte ratios for our Irish NAC-treated cohort (including subtype-specific values) we aim to provide the field with fundamental values/ranges that others can build upon to set a clinically relevant benchmark or standard with which to compare and evaluate other cohorts.

## 5. Conclusions

In this study, we examine the prognostic value of immunocyte ratios in NAC-treated breast cancer patients. Our results show that there is significant variation in the application of specific immunocyte ratios by breast cancer subtype. This is consistent with our advancing understanding of the molecular differences demonstrated to underpin each breast cancer subtype. This work reinforces the need to fully understand how the subtype-specific molecular differences manifest to alter systemic clinically relevant features, highlights the need for a large multi-national study with a longer follow-up to examine the range and variation of subtype-specific immunocyte ratios to validate their use as clinically relevant prognostic markers in neoadjuvant chemotherapy-treated breast cancer.

## Figures and Tables

**Table 1:** Patient characteristics by cancer subtypes (clinical data).

Characteristic	*N*	Overall *N* = 353 ^1^	Luminal A *N* = 108 ^1^	Luminal B *N* = 122 ^1^	HER2-Positive *N* = 80 ^1^	Triple Negative *N* = 43 ^1^	*p*-Value ^2^
Overall survival, months (range)	353	45.0 (25.0, 67.0)	32.5 (14.0, 48.0)	49.0 (33.0, 72.8)	52.0 (27.5, 72.0)	51.0 (34.0, 67.0)	<0.001
Disease-free survival months (range)	353	40.0 (18.0, 65.0)	28.5 (12.0, 44.0)	45.5 (27.8, 71.0)	46.5 (13.8, 65.8)	51.0 (27.5, 67.0)	<0.001
Recurrence	353						0.041
No		288.0 (81.6%)	83.0 (28.8%)	107.0 (37.2%)	60.0 (20.8%)	38.0 (13.2%)	
Yes		65.0 (18.4%)	25.0 (38.5%)	15.0 (23.1%)	20.0 (30.8%)	5.0 (7.7%)	
Age	353	55.0 (46.0, 68.0)	47.5 (41.0, 56.2)	62.0 (51.0, 72.0)	62.0 (53.8, 71.0)	46.0 (40.5, 55.5)	<0.001
Tumor size, in mm (range)	200	22.0 (12.0, 30.2)	NA	22.0 (15.0, 31.2)	21.5 (9.2, 30.0)	NA	0.31
Tumor grade (percent)	353						
0		14.0 (4.0%)	3.0 (21.4%)	4.0 (28.6%)	6.0 (42.9%)	1.0 (7.1%)	
1		7.0 (2.0%)	7.0 (100.0%)	0.0 (0.0%)	0.0 (0.0%)	0.0 (0.0%)	
2		136.0 (38.5%)	65.0 (47.8%)	53.0 (39.0%)	11.0 (8.1%)	7.0 (5.1%)	
3		196.0 (55.5%)	33.0 (16.8%)	65.0 (33.2%)	63.0 (32.1%)	35.0 (17.9%)	
Adjuvant chemotherapy	338						
Yes		137.0 (40.5%)	0.0 (0.0%)	82.0 (59.9%)	55.0 (40.1%)	0.0 (0.0%)	
No		36.0 (10.65%)	0.0 (0.0%)	23.0 (63.9%)	13.0 (36.1%)	0.0 (0.0%)	
Unknown		165.0 (48.8%)	108.0 (65.5%)	9.0 (5.5%)	5.0 (3.0%)	43.0 (26.1%)	
Metastasis	353						
No		190.0 (53.8%)	0.0 (0.0%)	118.0 (62.1%)	72.0 (37.9%)	0.0 (0.0%)	
Yes		12.0 (3.4%)	0.0 (0.0%)	4.0 (33.3%)	8.0 (66.7%)	0.0 (0.0%)	
Unknown		151.0 (42.8%)	108.0 (71.5%)	0.0 (0.0%)	0.0 (0.0%)	43.0 (28.5%)	

^1^ Median (IQR); *n* (%). ^2^ Kruskal–Wallis rank sum test; Pearson’s chi-squared test.

**Table 2:** Cohort immunocyte counts.

Characteristic	*N*	Overall, *N* = 353 ^1^	Luminal A *N* = 108 ^1^	Luminal B *N* = 122 ^1^	HER2-Positive *N* = 80 ^1^	Triple Negative *N* = 43 ^1^	*p*-Value ^2^
Total WCC	352	7.4 (6.0, 8.7)	6.6 (5.7, 8.6)	7.6 (6.0, 8.7)	7.7 (6.5, 8.8)	7.6 (6.4, 8.9)	0.082
Red blood cell count (RBC) %	353	4.5 (4.2, 4.7)	4.5 (4.2, 4.7)	4.5 (4.2, 4.8)	4.4 (4.2, 4.7)	4.5 (4.2, 4.7)	0.72
Hemoglobin	353	13.4 (12.6, 14.1)	13.4 (12.7, 14.0)	13.4 (12.6, 14.3)	13.2 (12.5, 13.9)	13.4 (12.5, 14.0)	0.59
Hematocrit	353	0.4 (0.4, 0.4)	0.4 (0.4, 0.4)	0.4 (0.4, 0.4)	0.4 (0.4, 0.4)	0.4 (0.4, 0.4)	0.45
Neutrophils #, 10^3^/μL	353	4.7 (3.5, 5.9)	4.0 (3.2, 5.7)	4.9 (3.6, 5.9)	4.8 (3.9, 5.6)	5.2 (4.0, 6.0)	**0.025**
Lymphocytes	353	1.8 (1.4, 2.3)	1.7 (1.4, 2.1)	1.9 (1.4, 2.4)	2.0 (1.6, 2.5)	1.6 (1.3, 2.0)	0.089
Monocytes #, 10^3^/μL	353	0.4 (0.3, 0.5)	0.4 (0.3, 0.5)	0.4 (0.3, 0.4)	0.4 (0.3, 0.5)	0.4 (0.3, 0.5)	0.79
Eosinophils #, 10^3^/μL	353	0.2 (0.1, 0.2)	0.2 (0.1, 0.3)	0.1 (0.1, 0.2)	0.2 (0.1, 0.3)	0.1 (0.1, 0.2)	**0.023**
Basophils #, 10^3^/μL	353						0.52
0–0.09		211.0 (59.8%)	65.0 (30.8%)	72.0 (34.1%)	44.0 (20.9%)	30.0 (14.2%)	
0.1–0.59		141.0 (39.9.0%)	42.0 (29.8%)	50.0 (35.5%)	36.0 (25.5%)	13.0 (9.2%)	
>0.6		1.0 (0.3%)	1.0 (100.0%)	0.0 (0.0%)	0.0 (0.0%)	0.0 (0.0%)	

^1^ Median (IQR); *n* (%). ^2^ Kruskal–Wallis rank sum test; Fisher’s exact test. # counts at 10^3^/μL. Significant results highlighted in Bold.

**Table 3:** Median systemic immunocyte ratios.

Characteristic	*N*	Overall *N* = 353 ^1^	Luminal A *N* = 108 ^1^	Luminal B *N* = 122 ^1^	Her2 Positive *N* = 80 ^1^	Triple Negative *N* = 43 ^1^	*p*-Value ^2^
NLR	353	2.5 (1.8, 3.5)	2.3 (1.7, 3.3)	2.6 (1.9, 3.4)	2.3 (1.7, 3.3)	2.8 (2.2, 4.3)	0.092
LMR	353	5.0 (3.5, 6.7)	4.6 (3.2, 6.7)	5.3 (4.0, 6.7)	5.0 (3.7, 7.0)	4.8 (3.0, 6.0)	0.19
NWR	353	0.6 (0.6, 0.7)	0.6 (0.6, 0.7)	0.7 (0.6, 0.7)	0.6 (0.6, 0.7)	0.7 (0.6, 0.7)	0.070
LWR	353	0.3 (0.2, 0.3)	0.3 (0.2, 0.3)	0.3 (0.2, 0.3)	0.3 (0.2, 0.3)	0.2 (0.2, 0.3)	0.15
MWR	353	0.1 (0.0, 0.1)	0.1 (0.0, 0.1)	0.1 (0.0, 0.1)	0.1 (0.0, 0.1)	0.1 (0.0, 0.1)	0.21

^1^ Median (IQR). ^2^ Kruskal–Wallis rank sum test.

**Table 4:** Correlations between clinicopathological details and immunocyte ratios.

	*p*-Value (Correlation)				
	NLR	LMR	NWR	LWR	MWR
Luminal A (*n* = 108)					
OS *	0.599 (0.05)	0.232 (0.12)	0.710 (0.04)	0.829 (−0.02)	**0.010 (−0.25)**
DFS *	0.918 (0.01)	0.250 (0.11)	0.946 (0.01)	0.874 (0.02)	**0.018 (−0.23)**
Tumor grade **	0.494	0.339	0.495	0.219	0.788
Luminal B (*n* = 122)					
OS *	0.167 (−0.13)	**0.018 (0.21)**	0.575 (−0.05)	0.114 (0.14)	**0.014 (−0.22)**
DFS *	0.169 (−0.13)	**0.005 (0.25)**	0.487 (−0.06)	0.088 (0.15)	**0.009 (−0.24)**
Tumor grade **	0.155	0.265	0.100	0.557	0.204
HER2-positive (*n* = 80)					
OS *	**0.018 (−0.26)**	0.086 (0.19)	**0.005 (−0.31)**	**0.006 (0.30)**	0.774 (0.03)
DFS *	**0.006 (−0.30)**	0.223 (0.14)	**0.001 (−0.36)**	**0.003 (0.32)**	0.442 (0.09)
Tumor grade **	0.978	0.212	0.482	0.277	0.725
Triple negative (*n* = 43)					
OS *	0.575 (−0.09)	0.442 (0.12)	0.757 (−0.05)	0.567 (−0.09)	0.108 (−0.25)
DFS *	0.463 (−0.11)	0.316 (0.16)	0.646 (−0.07)	0.702 (−0.06)	0.083 (−0.27)
Tumor grade **	0.992	0.260	0.961	0.821	0.089
**Combined cohort (*n* = 353)**					
OS *	0.138 (−0.08)	**0.002 (0.16)**	0.327 (−0.05)	0.143 (0.08)	**0.005 (−0.15)**
DFS *	**0.038 (−0.11)**	**0.002 (0.16)**	0.125 (−0.08)	0.053 (0.10)	**0.015 (−0.13)**
Tumor grade **	0.224	0.364	**0.029**	0.068	0.480

* *p*-value (correlation). ** ANOVA test *p*-value. Significant results highlighted in Bold.

**Table 5:** Optimal immunocyte ratio cut-off values by cancer subtype for 3-year OS.

	Cut Off * (Sensitivity, Specificity)				
	NLR	LMR	NWR	LWR	MWR
Luminal A	<1.53 (80.0%, 12.5%)	<7.17 (81.7%, 20.8%)	<0.54 (80.0%, 16.7%)	<0.20 (81.7%, 18.8%)	<0.035 (85.0%, 18.8%)
Luminal B	<1.96 (80.6%, 32.6%)	<5.69 (80.6%, 46.5%)	<0.60 (80.6%, 36.0%)	<0.30 (80.6%, 31.4%)	<0.040 (80.6%, 20.9%)
HER2-positive	<1.82 (80.0%, 34.5%)	<6.29 (80.6%, 46.5%)	<0.59 (80.0%, 41.8%)	<0.33 (80.0%, 32.7%)	<0.039 (80.0%, 18.2%)
Triple negative	<2.31 (83.3%, 35.5%)	<2.08 (83.3%, 6.5%)	<0.62 (83.3%, 35.5%)	<0.28 (83.3%, 29.0%)	<0.035 (83.3%, 9.7%)
Combined	<1.70 (80.5%, 20.9%)	<6.71 (82.0%, 27.3%)	<0.56 (80.5%, 19.1%)	<0.33 (80.5%, 21.4%)	<0.04 (81.2%, 18.6%)

* Cut-off points are chosen in a way so as to ensure at least 80% sensitivity.

**Table 6:** Optimal immunocyte ratio cut-off values by cancer subtype for 3-year DFS.

	Cut Off * (Sensitivity, Specificity)				
	NLR	LMR	NWR	LWR	MWR
Luminal A	<1.59 (80.0%, 14.0%)	<7.17 (80.0%, 18.6%)	<0.54 (81.5%, 18.6%)	<0.35 (80.0%, 11.6%)	<0.035 (80.0%, 11.6%)
Luminal B	<1.96 (81.0%, 33.8%)	<5.69 (81.0%, 48.8%)	<0.60 (81.0%, 37.5%)	<0.30 (81.0%, 32.5%)	<0.040 (81.0%, 21.3%)
HER2-positive	<1.77 (82.4%, 34.8%)	<7.38 (82.4%, 26.1%)	<0.58 (82.4%, 37.0%)	<0.33 (82.4%, 32.6%)	<0.039 (82.4%, 15.2%)
Triple negative	<2.31 (85.7%, 37.9%)	<6.59 (85.7%, 20.7%)	<0.61 (85.7%, 31.0%)	<0.28 (85.7%, 31.0%)	<0.035 (85.7%, 10.3%)
Combined	<1.79 (80.0%, 25.2%)	<6.71 (80.6%, 27.3%)	<0.57 (81.3%, 24.2%)	<0.32 (80.6%, 23.2%)	<0.04 (80.6%, 16.7%)

* Cut-off points are chosen in such a way as to ensure at least 80% sensitivity.

**Table 7:** Immunocyte ratio cut-off correlation with OS.

	*p*-Value (Cutoff *; # of Low-Marker Values, and # of High-Marker Values)				
	NLR	LMR	NWR	LWR	MWR
Luminal A	0.969 (1.79; 31 and 77)	**0.004** **(5.29; 68 and 40)**	0.761 (0.56; 33 and 75)	0.836 (0.30; 73 and 35)	**0.022** **(0.06; 72 and 36)**
Luminal B	0.760 (1.79; 23 and 99)	**0.027** **(5.29; 61 and 61)**	0.795 (0.56; 22 and 100)	0.622 (0.30; 88 and 34)	**0.008** **(0.06; 92 and 30)**
HER2-positive	**0.013** **(1.79; 22 and 58)**	0.111 (5.29; 42 and 38)	0.380 (0.56; 14 and 66)	**0.043** **(0.30; 51 and 29)**	0.541 (0.06; 60 and 20)
Triple negative	0.169 (1.79; 5 and 38)	0.498 (5.29; 25 and 18)	0.430 (0.56; 3 and 40)	0.503 (0.30; 36 and 7)	0.330 (0.06; 33 and 10)
Combined	0.419 (1.79; 81 and 272)	**<0.001** **(5.29; 196 and 157)**	0.833 (0.56; 72 and 281)	0.349 (0.30; 248 and 105)	**0.001** **(0.06; 257 and 96)**

* Cut-off points are chosen in the way as to maximize the difference between the two groups using decision trees. # counts. Significant results highlighted in Bold.

**Table 8:** Immunocyte ratio cut-off correlation with DFS.

	*p*-Value (Cutoff *; # of Low-Marker Values, and # of High-Marker Values)				
	NLR	LMR	NWR	LWR	MWR
Luminal A	0.789 (1.79; 31 and 77)	**0.004** **(5.29; 68 and 40)**	0.380 (0.62; 54 and 54)	0.816 (0.30; 73 and 35)	**0.022** **(0.06; 72 and 36)**
Luminal B	0.586 (1.79; 23 and 99)	**0.005** **(5.29; 61 and 61)**	0.835 (0.62; 46 and 76)	0.517 (0.30; 88 and 34)	**0.007** **(0.06; 92 and 30)**
HER2-positive	**0.035** **(1.79; 22 and 58)**	0.202 (5.29; 42 and 38)	**0.021** **(0.62; 40 and 40)**	0.071 (0.30; 51 and 29)	0.250 (0.06; 60 and 20)
Triple negative	0.224 (1.79; 5 and 38)	0.401 (5.29; 25 and 18)	0.939 (0.62; 16 and 27)	0.570 (0.30; 36 and 7)	0.250 (0.06; 33 and 10)
Combined	0.447 (1.79; 81 and 272)	**<0.001** **(5.29; 196 and 157)**	0.218 (0.62; 156 and 197)	0.456 (0.30; 248 and105)	**0.004** **(0.06; 257 and 96)**

* Cut-off points are chosen in such a way as to maximize the difference between the two groups using decision trees. # counts. Significant results highlighted in Bold.

## Data Availability

The data presented in this study are available on reasonable request from the corresponding author.

## Supplementary Materials

The following supporting information can be downloaded at: https://www.mdpi.com/article/10.3390/curroncol29100591/s1, Figure S1: Model performances using the receiver operating characteristic (ROC) curves (top) and calibration curves (bottom). (A) Left column indicates OS model performances; (B) middle column indicates DFS model performances; and (C) right column indicates performances of tumor grade models.
