# Supplementary material for: Peripheral Blood Cell Ratios as Prognostic Indicators in a Neoadjuvant Chemotherapy-Treated Breast Cancer Cohort
Source: Curr Oncol. 2022 Oct 7;29(10):7512–23. doi: 10.3390/curroncol29100591 (PMC9600104; doi:10.3390/curroncol29100591)
Supplement: Supplementary file 1 [file curroncol-29-00591-s001.zip › Supplementary FIgures and Tables.pdf]

Supplementary Materials.

Multivariate analysis (Supplementary tables)

Modelling overall survival

- Cox model for OS with all biomarkers (i.e. NLR, LMR, NWR, LWR, MWR):

	HR[1]	95% CI[1]	p-value
NLR	1.03	0.93, 1.14	0.6
LMR	0.93	0.85, 1.01	0.081
NWR	1.78	0.16, 19.5	0.6
LWR	5.73	0.17, 198	0.3
MWR	4.34	0.12, 155	0.4

[1]HR = Hazard Ratio, CI = Confidence Interval

- Cox model for OS with the best combination of biomarkers (i.e. NLR, LMR, NWR, LWR, MWR):

	HR[1]	95% CI[1]	p-value
LMR	0.94	0.89, 0.98	0.008

[1]HR = Hazard Ratio, CI = Confidence Interval

- Cox models for OS including LMR and Tumour grade:

	HR[1]	95% CI[1]	p-value
Tumour grade			
2	—	—	
3	0.81	0.65, 1.02	0.068
LMR	0.93	0.88, 0.98	0.005

[1]HR = Hazard Ratio, CI = Confidence Interval

- Cox models for OS including LMR and Breast cancer subtype:

	HR[1]	95% CI[1]	p-value
Breast cancer subtype			
Luminal A	—	—	
Luminal B	0.73	0.56, 0.96	0.021
Her2 positive	0.74	0.55, 0.99	0.040
Triple negative	0.58	0.41, 0.83	0.003
LMR	0.93	0.89, 0.98	0.007

[1]HR = Hazard Ratio, CI = Confidence Interval

Supplemental Figure 1A

- Model performance for OS at 3-year - ROC curve:

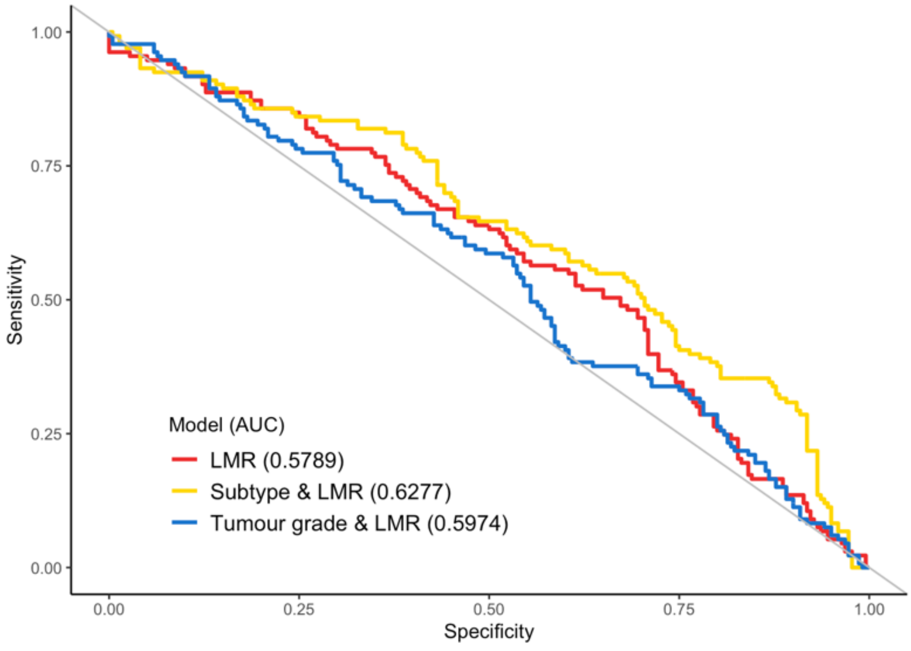

- Model performance for OS at 3-year - Calibration plot:

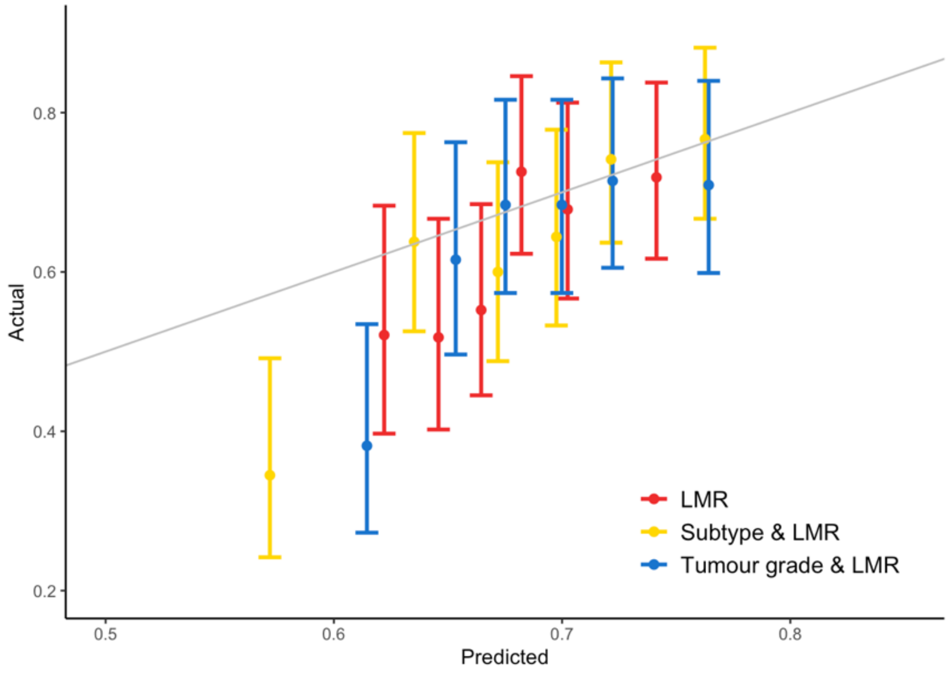

Modelling disease-free survival

- Cox model for DFS with all biomarkers (i.e. NLR, LMR, NWR, LWR, MWR):

	HR[1]	95% CI[1]	p-value
NLR	1.06	0.95, 1.19	0.3
LMR	0.92	0.85, 1.00	0.054
NWR	1.74	0.15, 19.8	0.7
LWR	9.09	0.24, 342	0.2
MWR	3.41	0.10, 113	0.5

[1]HR = Hazard Ratio, CI = Confidence Interval

- Cox model for DFS with the best combination of biomarkers (i.e. NLR, LMR, NWR, LWR, MWR):

Characteristic	HR[1]	95% CI[1]	p-value
LMR	0.93	0.88, 0.98	0.003

[1]HR = Hazard Ratio, CI = Confidence Interval

- Cox models for DFS including LMR and Distal metastasis sites:

	HR[1]	95% CI[1]	p-value
Distal metastasis sites			
No	—	—	
Yes	2.65	1.46, 4.81	0.001
Unknown	1.15	0.92, 1.43	0.2
LMR	0.93	0.88, 0.97	0.003

[1]HR = Hazard Ratio, CI = Confidence Interval

- Cox models for DFS including LMR, Distal metastasis sites and Breast cancer subtype:

	HR[1]	95% CI[1]	p-value
Distal metastasis sites			
No	—	—	
Yes	2.72	1.50, 4.95	0.001
Unknown	1.43	1.06, 1.93	0.018
Breast cancer subtype			
Luminal A	—	—	
Luminal B	0.97	0.73, 1.29	0.9
Her2 positive			
Triple negative	0.51	0.35, 0.73	<0.001
LMR	0.92	0.88, 0.97	0.001

[1]HR = Hazard Ratio, CI = Confidence Interval

Supplemental Figure 1B

- Model performance for DFS at 3-year - ROC curve:

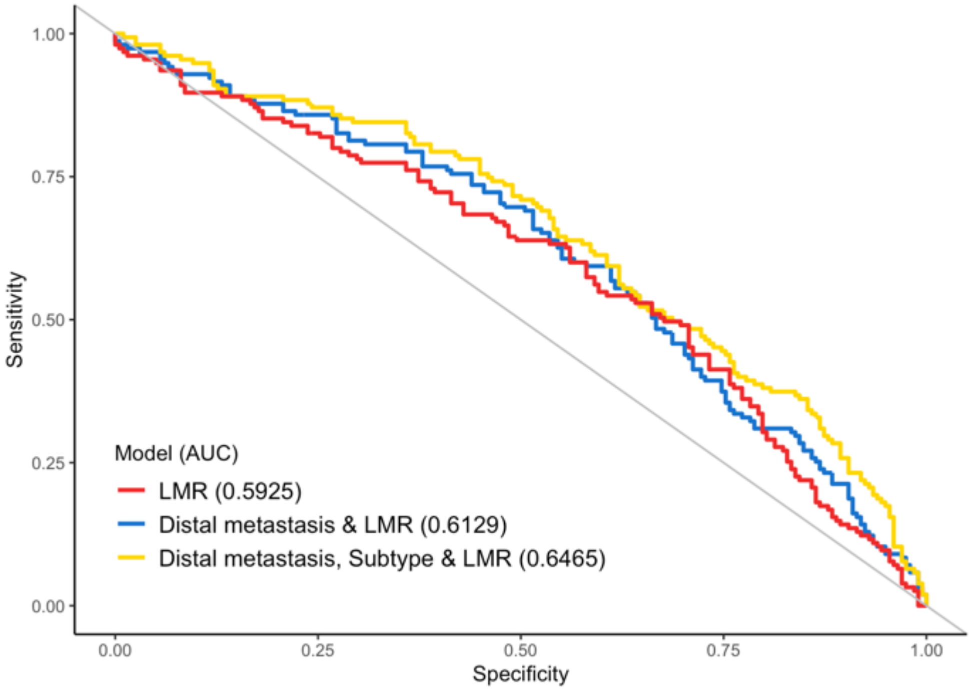

- Model performance for DFS at 3-year - Calibration plot:

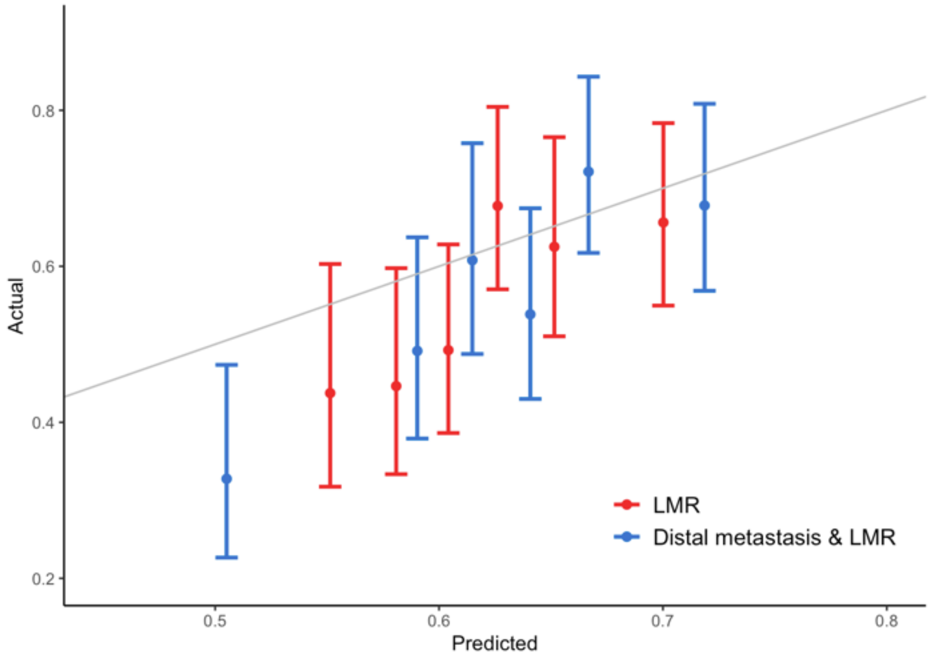

Modelling tumour grade:

There are 21 patients with tumour grade 0 and grade 1 that are excluded when modelling tumour grade, and models developed for predicting tumour grade 2 and grade 3.

- Logistic model for Tumour grade with all biomarkers (i.e. NLR, LMR, NWR, LWR, MWR):

	OR[1]	95% CI[1]	p-value
NLR	0.88	0.70, 1.06	0.2
LMR	0.99	0.83, 1.17	0.9
NWR	32.5	0.48, 11,928	0.14
LWR	3.55	0.01, 14,388	0.7
MWR	0.00	0.00, 56.2	0.3

[1]OR = Odds Ratio, CI = Confidence Interval

- Logistic model for Tumour grade with the best combination of biomarkers (i.e. NLR, LMR, NWR, LWR, MWR):

	OR[1]	95% CI[1]	p-value
NLR	0.86	0.70, 1.02	0.10
NWR	25.6	0.84, 1,067	0.072

[1]OR = Odds Ratio, CI = Confidence Interval

- Logistic model for Tumour grade including the best combination of biomarkers and clinical variables (i.e. Age, Distal metastasis sites, Breast cancer subtype):

	OR[1]	95% CI[1]	p-value
Breast.Cancer.Subtype			
Luminal A	—	—	
Luminal B	2.26	1.30, 3.99	0.004
Her2 positive	11.5	5.45, 26.2	<0.001
Triple negative	10.3	4.25, 28.2	<0.001
NLR	0.84	0.69, 1.02	0.072
NWR	13.8	0.36, 659	0.2
MWR	0.00	0.00, 6.45	0.14

[1]OR = Odds Ratio, CI = Confidence Interval
